# A novel circuit overrides Adr 1p control during expression of *Saccharomyces cerevisiae* 2-*trans*-enoyl-ACP reductase Etr 1p of mitochondrial type 2 fatty acid synthase

**DOI:** 10.1111/j.1574-6968.2009.01688.x

**Published:** 2009-07-03

**Authors:** Aner Gurvitz

**Affiliations:** Section of Physiology of Lipid Metabolism, Center for Physiology, Pathophysiology and Immunology, Institute of Physiology, Medical University of ViennaVienna, Austria

**Keywords:** Adr1p-binding site, type 1 upstream activation sequence UAS1, mitochondrial FASII, lipoic acid, transcriptional regulation, translational control

## Abstract

The significance of the chronicled role of the yeast transcription factor Adr1p in regulating *ETR1* was examined in wild type and isogenic *adr1*Δ mutant cells. An *ETR1-lacZ* reporter construct was used to verify Adr1p-dependent gene expression. On solid glycerol medium containing X-gal, wild-type cells expressing the reporter turned blue, whereas the *adr1*Δ mutants remained white. β-Galactosidase activity measurements following 24-h cell growth in liquid glycerol medium revealed a 6.5-fold greater expression level of the reporter gene in the wild type compared with the *adr1*Δ mutant. In contrast, immunoblotting showed that Etr1p abundance was essentially indistinguishable between the two strains whereas Cta1p, whose expression depends on Adr1p, was present in the wild-type cells, but not in the mutants. Moreover, enzyme assays conducted on transformed wild-type and *adr1*Δ mutant cells expressing a plasmid-borne *ETR1* tethered behind the native promoter revealed similar levels of reductase activity, and the lipoic acid content in the two parental strains was equivalent. Hence, while Adr1p influenced the transcription levels of *ETR1*, it did not alter the abundance of Etr1p, the level of reductase activity, or the cellular amount of lipoic acid. The results point toward a potentially novel layer of control for maintaining physiological levels of lipoic acid.

## Introduction

*De novo* fatty acid biosynthesis is a spiral process in which the nascent hydrocarbon chain is extended by two carbons per turn. Two types of eukaryotic fatty acid synthases (FAS) can undertake this procedure, their classification depending on the organization of their respective structures. In the associative type 1 system (FASI), the enzyme activities are represented by domains on a single cytoplasmic synthase, whereas in the compartmentalized type 2 system (FASII), each of these activities is dissociated into discrete proteins. FASII has been regarded as typically prokaryotic, with the *Escherichia coli* process having been described in detail (Rock & Cronan, 1996; White *et al*., 2005;). On the other hand, the existence of mitochondrial FASII in *Saccharomyces cerevisiae* is a relatively recent discovery (Hiltunen *et al*., 2005).

The precise product(s) of yeast mitochondrial FASII, other than lipoic acid, have not yet been positively identified, but it is now clear that a functional FASII is critical for the physiological activity of the organelle. A lesion in any one of the genes encoding FASII proteins results in respiratory deficiency; mutant cells fail to grow on nonfermentable carbon sources, contain only small mitochondria, and do not form cytochrome complexes or produce lipoic acid. Although all of the yeast FASII genes have been revealed (Hiltunen *et al*., 2005), including *ETR1* (Torkko *et al*., 2001), not much is known about their transcriptional regulation.

The *ETR1* promoter controlling the expression of 2-*trans*-enoyl-ACP reductase Etr1p contains a nucleotide sequence situated 91 bp upstream of the ATG triplet for the translational start codon, which is almost identical to the consensus sequence for an Adr1p-binding site (Young *et al*., 2003), termed type 1 upstream activation sequence UAS1, i.e. CYCCR(A/T/G)N4–36(T/A/C)YGGRG (Cheng *et al*., 1994). DNA microarray analysis performed previously on cells grown under derepressing conditions revealed that the expression of 108 genes, among them *ETR1*, is significantly decreased in the absence of Adr1p (Young *et al*., 2003). Moreover, it was shown that the *ETR1* promoter contains a functional UAS1 that complexes with the cognate transcription factor *in vivo* (Young *et al*., 2003). Here, the extent of the influence exerted by Adr1p on *ETR1* was scrutinized in wild-type and *adr1*Δ mutant cells using an *ETR1-lacZ* reporter gene, and LacZ expression was monitored in cells grown on solid and liquid media. The abundance of Etr1p in wild-type and *adr1*Δ mutant cells grown on glycerol medium was investigated by immunoblotting as well as reductase activity assays, and the effect of deleting *ADR1* on lipoic acid biosynthesis was monitored. The results are discussed in terms of a putative mechanism restricting the amount of lipoic acid in the mitochondria of wild-type cells despite the Adr1p-dependent signal to exit glucose repression.

## Materials and methods

### Yeast strains and plasmids

*Saccharomyces cerevisiae* strains and plasmids used are listed in Table 1. The *E. coli* strains DH10B and TOP10 F′ were used for plasmid amplifications and isolations. *Saccharomyces cerevisiae* strain BJ1991 (Jones, 1977) was described previously, and strains BY4741 wild type and BY4741*adr1*Δ were acquired from EUROSCARF (http://web.uni-frankfurt.de/). The plasmid vectors used, YCplac22, YCplac33, and YEplac195, are described (Gietz & Sugino, 1988). Plasmid YCp173 represented a YCplac33 centromeric vector carrying the nucleotide sequence for *lacZ* corresponding to *E. coli*β-galactosidase driven by 975 nucleotides of the *ETR1* (*MRF1*′) promoter and will be described elsewhere. Plasmid YEplac195–MRF1′ expressing *ETR1* behind the native promoter on a *URA3*-marked multicopy vector was constructed by applying PCR to yeast genomic DNA using oligonucleotides that amplified the *ETR1* gene with additional 0.97 kb upstream and 0.32 kb downstream sequences, and inserting the amplicon into a HindIII- and BamHI-digested YEplac195 vector. A similar strategy was used to generate the low-copy centromeric plasmid YCplac22-MRF1′. Plasmids were introduced into yeast cells using a published method (Chen *et al*., 1992).

**Table 1:** *Saccharomyces cerevisiae* strains and plasmids used

	Description	Sources or references
Strains		
BJ1991	*MAT*α*leu2 ura3-52 trp1 pep4-3 prb1-122 gal2*	Jones (1977)
BJ1991*adr1*Δ	*adr1*Δ::*LEU2*	Tabak Lab*
BY4741	*MAT***a***his3*Δ*1leu2*Δ*0met15*Δ*0ura3*Δ*0*	EUROSCARF
BY4741*adr1*Δ	*ydr216w*::*kanMX4*	EUROSCARF
Plasmids		
YCplac22	Yeast low-copy centromeric plasmid vector marked with *TRP1*	Gietz & Sugino (1988)
YCplac22-MRF1′	Loaded with *ETR1* flanked by the native promoter and terminator	Hiltunen Lab†
YCplac33	Yeast low-copy centromeric plasmid vector marked with *URA3*	Gietz & Sugino (1988)
YCp173	YCplac33 loaded with an *ETR1-lacZ* fusion	Hiltunen Lab
YEplac195	Yeast multicopy episomal plasmid vector marked with *URA3*	Gietz & Sugino (1988)
YEplac195-MRF1′	Loaded with *ETR1* flanked by the native promoter and terminator	Hiltunen Lab

* Dr H.F. Tabak, Department of Biochemistry, Academic Medical Center of the University of Amsterdam, the Netherlands.

† Dr J.K. Hiltunen, Department of Biochemistry and Biocenter Oulu, University of Oulu, Finland.

### Media and growth conditions

Standard yeast (Rose *et al*., 1990) and *E. coli* (Sambrook *et al*., 1989) media were made as described. YPD medium consisted of 1% (w/v) yeast extract, 2% (w/v) peptone (YP), 2% (w/v) d-glucose, and 2% (w/v) agar. In YPglycerol medium, glucose was replaced by 3% (w/v) glycerol as the sole carbon source. Liquid YPD and YPglycerol media were prepared essentially the same way, except that agar was omitted. *URA3*-marked plasmids were maintained in transformed strains using solid synthetic defined SD-Ura medium consisting of a 0.67% (w/v) yeast nitrogen base without amino acids, 2% (w/v) d-glucose, and 2% (w/v) agar, with all supplements added, except for uracil (Sigma-Aldrich Inc., MO). For enzyme assays, cells were grown overnight in liquid SD-Ura medium lacking agar, transferred to liquid YPglycerol medium, and cultured for a further 24-h period.

### Miscellaneous

The following two procedures were performed according to published methods: determination of protein concentrations (Bradford, 1976) and electrophoresis (Laemmli, 1970). β-Galactosidase activities were determined on solid media that contained 50 μL of 4% (w/v) 5-bromo-4-chloro-3-indolyl-β-d-galactopyranoside (X-gal), or assayed in soluble protein extracts that were prepared by breaking cells with glass beads, and expressed as nanomoles of *o*–nitrophenyl-β-d-galactopyranoside (ONPG) hydrolyzed per minute per milligram of protein (Miller, 1972). The measurements were conducted over the period of *c*. 1 h. Catalase measurements were performed as described (Beers & Sizer, 1952), and the values reported here are the average of three experiments±SD. Whole-cell extracts were prepared according to a published protocol (Yaffe & Schatz, 1984). Immunoreactive complexes were visualized using anti-rabbit immunoglobulin G-coupled horseradish peroxidase in combination with the enhanced chemiluminescence (ECL) system from Amersham Pharmacia Biotech. Enoyl reductase activity was assayed spectrophotometrically at 23 °C as described (Dommes & Kunau, 1984). The assay mixture consisted of 50 mM KP_i_ (pH 7.5) and 0.1 mg mL^−1^ bovine serum albumin, 125 μM NADPH, and 60 μM 2-*trans*-hexenoyl-CoA, which was synthesized via the mixed anhydride system (Goldman & Vagelos, 1961) as the substrate. Measurements of lipoic acid using the lipoic acid-deficient *E. coli* strain JRG33-lip9 have been described previously (Herbert & Guest, 1970; Hayden *et al*., 1993;).

## Results

### Adr 1p is necessary for elevated levels of *ETR1-lacZ* reporter activity

**Fig. 1:**
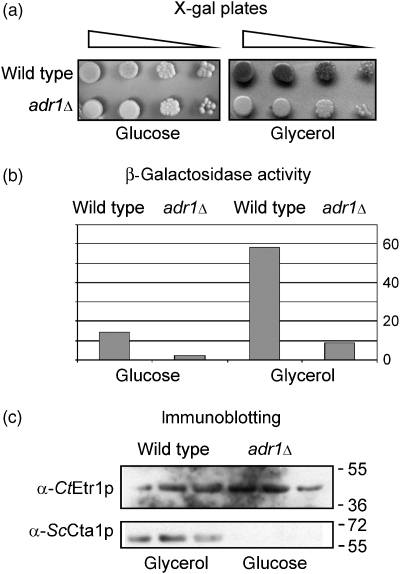
(a) Expression of an *ETR1-lacZ* reporter gene on glucose or glycerol media. Centromeric plasmid YCp173 (Table 1) was introduced into BY4741 wild-type cells or otherwise isogenic mutants devoid of Adr1p (*adr1*Δ). Tenfold serially diluted cultures (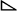) were applied to the indicated solid media containing X-gal. (b) The effect of the *adr1*Δ deletion on the expression of an *ETR1-lacZ* reporter gene in cells grown in liquid glycerol medium. BY4741 wild-type and *adr1*Δ mutant cells harboring YCp173 were grown for 2 days on synthetic glucose medium lacking uracil (SD-Ura) selecting for plasmid maintenance (0 h), and shifted to a rich glycerol medium (YPglycerol) for 24 h. β-Galactosidase activity was measured in soluble protein extracts, and the values obtained were as follows: 0 h wild type, 14.3; 0 h *adr1*Δ mutant, 2.4; 24 h wild type, 58.1±36.5; 24 h *adr1*Δ mutant, 9.0±3.9 (mean±SD, *n*=3). (c) Immunoblotting for the presence of Etr1p and Cta1p in BY4741 wild-type and *adr1*Δ mutant cells. The above extracts were subjected to 10% (w/v) sodium dodecyl sulfate-polyacrylamide gel electrophoresis (in duplicate), and resolved proteins were stained with Coomasie blue for verification of equal loading (not shown) or immobilized onto a nitrocellulose membrane. The membrane was incubated with an anti-Etr1p antibody raised against the homologous protein in *Candida tropicalis*, and the signal was visualized using the ECL system and recorded on an X-ray film. The membrane was sequentially washed and incubated with an anti-Cta1p antibody, and the signal was recorded as described above.

Previous genome-wide work in combination with chromatin immunoprecipitation demonstrated that Adr1p binds to the promoter of *ETR1 in vivo*, and that when propagated under derepressing conditions in YP medium containing 0.05% glucose, *adr1*Δ mutant cells expressed *ETR1* 3.2-fold less abundantly than the corresponding wild-type cells (Young *et al*., 2003). To examine the dependence of *ETR1* expression on Adr1p more closely, an *ETR1-lacZ* reporter loaded onto a centromeric plasmid was introduced into BY4741 wild-type and *adr1*Δ mutant cells. Transformants were grown overnight in liquid SD-Ura for plasmid maintenance, and following 10-fold serial dilution (starting with an A_600 nm_ of 1.0) were applied to solid media comprising YPD or YPglycerol that additionally contained X-gal, and the plates were incubated at 30 °C until the colonies turned blue (Fig. 1a). Although the aforementioned work demonstrated that cells devoid of Adr1p fail to grow on a synthetic complete medium containing glycerol, whereas wild-type cells grow abundantly on this medium, in the present study, both strains derived from the BY4741 genetic background grew extensively on YPglycerol. This observation was significant because it circumvented possible scenarios whereby differences in the levels of reporter-gene expression could have been due to an energy deficit in the mutant strain. In addition, the present results also confirmed that the wild-type strain efficiently expressed *ETR1* on glucose, and even more abundantly on glycerol, whereas the *adr1*Δ mutant was less efficient because it remained essentially white on both media (Fig. 1a).

To examine with greater precision the effect of deleting *ADR1* on the expression levels of *ETR1* in yeast cells, β-galactosidase measurements were undertaken using ONPG. As a control, the Adr1p-regulated *CTA1* gene (Simon *et al*., 1991; Young *et al*., 2002;) encoding peroxisomal catalase A was also analyzed. Catalase and β-galactosidase activities were assayed using soluble protein extracts obtained from liquid cultures grown on SD-Ura medium for 2 days, diluted to an A_600 nm_ of 0.2 in YPglycerol medium, and grown for a further 24-h period. The results of the β-galactosidase measurements showed that wild-type cells grown on YPglycerol produced 6.5-fold higher levels of the *lacZ* reporter compared with the *adr1*Δ mutant (Fig. 1b), whereas the outcome of the control catalase measurements, which additionally detected residual enzyme activity due to the Adr1p-independent cytosolic catalase Ctt1p, demonstrated that on YPglycerol, wild-type extracts gave rise to levels that were 1.6-fold higher compared with mutant extracts (wild type; 54±44; *adr1*Δ, 33±14; catalase activity values are units per mg protein and represent the mean±SD, *n*=3). Hence, *ETR1* transcription depended on Adr1p.

### Adr 1p does not influence Etr 1p abundance in wild-type cells

To compare the abundance of Etr1p within the two strains, immunoblotting was performed on the soluble protein extracts from the previous section. Duplicate gels were used to resolve the cellular protein contents, and one gel was stained with Coomasie blue to verify equal protein loading (not shown), whereas the other was used to transfer the resolved proteins onto a nitrocellulose membrane. The membrane was incubated with a primary anti-*Ct*Etr1p antibody that was raised in rabbits and directed against the homologous Etr1p protein in *Candida tropicalis*. This antibody was used previously to label thin sections for ultrastructural analysis of *S. cerevisiae* cells overexpressing either *C. tropicalis* or *S. cerevisiae* Etr1p (Torkko *et al*., 2001). Following application of the ECL system, the signals were recorded on an X-ray film. The results demonstrated the presence of an Etr1p signal of the correct size in the triplicate samples of each of the two cell types (Fig. 1c; upper panel). As a positive control, the presence of the aforementioned Adr1p-dependent Cta1p was also monitored in these samples. The same membrane was washed clean of the previous antibody, and a fresh anti-Cta1p antibody was applied. The results showed that the signal for Cta1p was evident in the wild type, but was missing from extracts obtained from the *adr1*Δ mutant (Fig. 1c; lower panel). Hence, the results indicated that unlike the situation with the Cta1p signal, whose intensity in wild-type extracts was elevated due to Adr1p, this was not the case with the signal attributable to Etr1p because the latter's intensity in the wild type did not exceed that in the mutant.

### Wild-type and *adr 1*Δ cells contain similar levels of reductase activities

The observation of equal amounts of Etr1p in both cells types was unexpected, and so, despite the chronicled specificity of the antibody for *S. cerevisiae* Etr1p (Torkko *et al*., 2001), a further method for monitoring the levels of this protein in yeast cells was adopted. Spectrophotometric enzyme assays for 2-*trans*-enoyl-CoA reductase activity offer an attractive alternative to immunoblotting for determining levels of active enzyme in soluble protein extracts. However, because native Etr1p levels are too low to be detected in this assay, a *URA3*-marked multicopy plasmid YEplac195-MRF1′ (Table 1) was used to amplify the reductase activity. In this plasmid construct, the gene for Etr1p is flanked by the native promoter and terminator sequences, and hence was expected to be expressed similarly to *ETR1* at the genomic locus.

BJ1991 wild type or *adr1*Δ mutants were transformed to uracil prototrophy with YEplac195-MRF1′, and following 24-h growth in YPglycerol medium, the cells were harvested and soluble protein extracts were prepared. Volumes of 0.5-μL protein extracts were mixed into a reaction mixture consisting of phosphate buffer and NADPH, and following the addition of 2-*trans*-hexenoyl-CoA as substrate, the progress of dinucleotide oxidation was monitored using a spectrophotometer. The results, expressed in micromoles of substrate reduced per minute per milligram of protein, showed that *adr1*Δ mutant cells producing Etr1p from the multicopy plasmid contained an activity of 6.4±2.7 (mean±SD, *n*=3) whereas wild-type extracts gave rise to an activity level commensurate with 4.4±2.71.5 (mean±SD, *n*=3). To emulate more physiological levels of Etr1p expression, enzyme assays were also conducted on strains expressing this *ETR1* construct from a low-copy centromeric plasmid YCplac22-MRF1′; however, these strains produced activities that were below the detection limit of the assay used. Hence, the absence from an enhanced level of 2-*trans*-enoyl-CoA reductase activity in the wild-type strain correlated with the previous immunoblotting result, indicating that Etr1p was neither more ample, nor active in the wild type compared with the situation in the *adr1*Δ mutant.

### Ample lipoic acid biosynthesis in wild-type and *adr 1*Δ mutant cells

If Etr1p is indeed present to the same extent in both strains, it follows that lipoic acid biosynthesis would also be comparable between them. Levels of lipoic acid were determined (in duplicate) in wild-type and *adr1*Δ mutant cells derived from two genetic backgrounds, BJ1991 and BY4741, and the values were expressed as nanograms of lipoic acid per gram wet weight of cells. The assay showed no meaningful difference in the lipoic acid content among the four strains that were examined (BJ1991 wild type, 236; BJ1991*adr1*Δ, 237; BY4741 wild type, 229; BY4741*adr1*Δ, 233). The significance of the combined results is discussed below.

## Discussion

In the present study, the issue of Etr1p abundance in yeast cells was approached using three different methods, and this revealed that despite *ETR1* transcription being governed by Adr1p, at the protein level, Etr1p was not more abundant in wild-type cells than in those lacking the transcription factor. The observations made here were (1) immunoblotting failed to reveal an increased abundance of Etr1p in protein extracts obtained from verifiably derepressed wild-type cells compared with those produced from the *adr1*Δ mutant, (2) reductase assays relying on ectopic Etr1p expression presumed to obey Adr1p regulation detected similar levels of enzyme activity in wild-type and *adr1*Δ extracts, and (3) physiological quantities of lipoic acid were comparable between the wild type and *adr1*Δ mutant using two different strain types. Because of the present results with the *ETR1-lacZ* reporter construct, there is no refuting the previous discovery relating to the *in vivo* binding of Adr1p to the *ETR1* promoter or the observation that *adr1*Δ mutant cells express *ETR1* less abundantly compared with wild-type cells (Young *et al*., 2003). However, reasonable doubt has now been cast as to whether transcriptional regulation exerted by Adr1p represents the sole – or for that matter – the most meaningful layer of control over Etr1p abundance in wild-type cells.

The reason why Etr1p expression is held back in the wild type is not yet clear. Nevertheless, it is attractive to speculate that this might be due to the potentially deleterious effects of abnormally high levels of lipoic acid or any other presumed FASII end products to the integrity of the mitochondrial compartment. Previous nonphysiological amplification of Etr1p levels obtained by tethering *ETR1* to the promoter of the oleic acid-inducible gene *CTA1* and growing cells on oleic acid resulted in yeast mitochondria that were both fewer in number and larger in size (Torkko *et al*., 2001). Thus, it would be interesting to determine whether this mitochondrial abnormality would also be reproducible in cells hosting the aforementioned plasmid expressing *ETR1* fitted between the native promoter and terminator sequences. A feature of Etr1p that has hitherto remained underappreciated is that it is also a nuclear protein that preferentially binds to the T-rich strand forming the core of the autonomous replication sequence (Yamazoe *et al*., 1994). Hence, the inherent possibility of regulation by Etr1p of its own translation must be considered in future work.

## Statement

Reuse of this article is permitted in accordance with the terms and conditions set out at http://www3.interscience.wiley.com/authorresources/onlineopen.html
